# Supplementary material for: Qubit readout error mitigation with bit-flip averaging
Source: Sci Adv. 2021 Nov 17;7(47):eabi8009. doi: 10.1126/sciadv.abi8009 (PMC8598004; doi:10.1126/sciadv.abi8009)
Supplement: Supplementary file 1 — Supplementary Text Tables S1 to S3 [file sciadv.abi8009_sm.pdf]

Science Advances
AAAS

Supplementary Materials for

Qubit readout error mitigation with bit-flip averaging

Alistair W. R. Smith*, Kiran E. Khosla, Chris N. Self, M. S. Kim

*Corresponding author. Email: alistair.smith18@imperial.ac.uk

Published 17 November 2021, *Sci. Adv.* **7**, eabi8009 (2021)
DOI: 10.1126/sciadv.abi8009

This PDF file includes:

Supplementary Text
Tables S1 to S3

Supplementary Text

I. EXAMPLE OF MITIGATION WITH SPARSE REPRESENTATION

Here we give an illustrative example of how BFA can simplify readout error mitigation. Suppose we have a four-qubit system in which errors on two qubits are completely correlated (i.e. when an error occurs on one qubit it always occurs on the other). An example of the underlying response matrix for this system could be:

$$\mathbf{M} = \underbrace{\begin{pmatrix} 0.98 & 0.08 \\ 0.02 & 0.92 \end{pmatrix}}_{\text{qubit 3}} \otimes \underbrace{\begin{pmatrix} 0.96 & 0 & 0 & 0.16 \\ 0 & 0.94 & 0.1 & 0 \\ 0 & 0.06 & 0.9 & 0 \\ 0.04 & 0 & 0 & 0.84 \end{pmatrix}}_{\text{qubit 2 and 1}} \otimes \underbrace{\begin{pmatrix} 0.97 & 0.11 \\ 0.03 & 0.89 \end{pmatrix}}_{\text{qubit 0}} \tag{S1}$$

Under BFA the symmetrised form of this matrix (using the sparse representation in Eq. 9) is:

$$\begin{aligned} \widetilde{\mathbf{M}} &= \underbrace{(0.95\mathbb{1} + 0.05X)}_{\text{qubit 3}} \otimes \underbrace{(0.91\mathbb{1}\mathbb{1} + 0.09XX)}_{\text{qubit 2 and 1}} \otimes \underbrace{(0.93\mathbb{1} + 0.07X)}_{\text{qubit 0}} \\ &= 0.804\mathbb{1}\mathbb{1}\mathbb{1}\mathbb{1} + 0.06\mathbb{1}\mathbb{1}\mathbb{1}X + 0.8\mathbb{1}XX\mathbb{1} \\ &\quad + 0.006\mathbb{1}XXX + 0.042X\mathbb{1}\mathbb{1}\mathbb{1} + 0.003X\mathbb{1}\mathbb{1}X \\ &\quad + 0.004XXX\mathbb{1} + 0.0003XXXX, \end{aligned} \tag{S2}$$

where, as discussed in section II A, the coefficients in front of the operators in this expansion are the probabilities that errors of a the corresponding syndrome occur (under bit-flipping). Suppose we now take a set of calibration measurements, inputting the state $|0000\rangle$, applying random bit-flips before a measurement and correcting the result to undo the bitflip. An example set of calibration results for this (with 10,000 repeats), showing the observed frequencies/probabilities for the different outcomes and their true values (from the underlying response matrix), is shown in Table I. This immediately provides us with an estimate $\widetilde{\mathbf{M}}^*$ for the BFA-simplified response matrix $\widetilde{\mathbf{M}}$ as

$$\begin{aligned} \widetilde{\mathbf{M}}^* = &\ 0.8091\mathbb{1}\mathbb{1}\mathbb{1}\mathbb{1} + 0.0595\mathbb{1}\mathbb{1}\mathbb{1}X + 0.784\mathbb{1}XX\mathbb{1} \\ &+ 0.0061\mathbb{1}XXX + 0.0433X\mathbb{1}\mathbb{1}\mathbb{1} + 0.0022X\mathbb{1}\mathbb{1}X \\ &+ 0.0046XXX\mathbb{1} + 0.0004XXXX. \end{aligned} \tag{S3}$$

This has an infidelity $(1 - \mathcal{F}_{\mathcal{M}})$ with the true response matrix of $\approx 10^{-4}$. However, this estimate does not necessarily exactly admit the decomposition in Eq. S2 (qubits 0 and 3 having independent errors and qubits 1 and 2 correlated errors). If we had prior knowledge that such a decomposition was likely to be correct then we could instead assume this decomposition and infer the probabilities by looking at the marginal results, shown in Table II.

BFA Simulated Calibration Results			
Outcome	Obs. freq.	Est. prob.	True prob.
0000	8091	0.8091	0.8040
0001	595	0.0595	0.0605
0110	784	0.0784	0.0795
0111	61	0.0061	0.0060
1000	433	0.0433	0.0423
1001	22	0.0022	0.0032
1110	46	0.0046	0.0042
1111	4	0.0004	0.0003

Table I. **Example simulated calibration results under BFA for the response matrix given in Eq. S1.** Shots (10,000) are sampled randomly from the columns in the underlying $\mathbf{M}$ (equivalent to bit-flipping an input state $|0\ldots0\rangle$) and a classical correction is applied.

Qubit 0 BFA Calibration Marginals			
Outcome	Obs. freq.	Est. prob.	True prob.
0	9318	0.9318	0.93
1	682	0.0682	0.07
Qubits 2 and 1 BFA Calibration Marginals			
Outcome	Obs. freq.	Est. prob.	True prob.
00	9141	0.9141	0.91
01	0	0.0	0.0
10	0	0.0	0.0
11	859	0.0859	0.09
Qubit 3 BFA Calibration Marginals			
Outcome	Obs. freq.	Est. prob.	True prob.
0	9495	0.9495	0.95
1	505	0.0505	0.05

Table II. **Marginal simulated calibration results under BFA for the response matrix in Eq. S1.** These results are those shown in Table I marginalised on the individual qubits 0 and 3 and the pair of qubits 1 & 2 that undergo correlated errors.

By making this assumption about the structure of the readout errors we reach a slightly different estimate for the response matrix:

$$\begin{aligned} \widetilde{\mathbf{M}}^* = \underbrace{(0.945\mathbb{1} + 0.05X)}_{\text{qubit 3}} \otimes \underbrace{(0.914\mathbb{1}\mathbb{1} + 0.0859XX)}_{\text{qubit 2 and 1}} \\ \otimes \underbrace{(0.9318\mathbb{1} + 0.0682X)}_{\text{qubit 0}}, \end{aligned} \tag{S4}$$

which has a lower infidelity of $\approx 3 \times 10^{-5}$ and so will result in more accurate mitigation. A tensor product structure such as this greatly reduces the computational cost of finding the inverse response matrix (which is the tensor product of the inverse matrices for qubit 3, qubits

2 and 1, and qubit 0 respectively). In this case keeping the more general structure in Eq. S3 also allows the inverse to be found more easily as the Pauli matrices in this expansion form a closed group under matrix multiplication. This means that $(\widetilde{\mathbf{M}}^*)^{-1}$ must contain the same set of X operators as $\widetilde{\mathbf{M}}^*$ $(\mathbb{1}\mathbb{1}\mathbb{1}\mathbb{1}, \mathbb{1}\mathbb{1}\mathbb{1}X$, etc.) meaning that fewer coefficients need to be calculated than for a generic $\widetilde{\mathbf{M}}^{-1}$.

The constrained optimization approach to mitigation is also made easier as the response matrix is relatively sparse. For measurements of some states this is made easier still as we can use a reduced set of possible outcomes. As an example we consider the noisy readout of a noiseless preparation of the 4-qubit GHZ state $(|0000\rangle + |1111\rangle)/\sqrt{2}$. If the underlying response matrix that in Eq. S1, a set of possible outcome measurements (probabilities from 10,000 shots) while using BFA is given in Table III.

BFA 4-qubit GHZ State Simulated Results			
Outcome	Observed prob.	Expected noisy prob.	Noiseless prob.
0000	0.4048	0.40215	0.5
0001	0.0330	0.03235	0.0
0110	0.0407	0.04135	0.0
0111	0.0243	0.02415	0.0
1000	0.0235	0.02415	0.0
1001	0.0414	0.04135	0.0
1110	0.0316	0.03235	0.0
1111	0.4007	0.40215	0.5

Table III. **Simulated GHZ state measurement results under BFA.** These probabilities are calculated from 10,000 shots. BFA is simulated by applying shot-wise random bit-flips to noiseless simulated results, sampling from the corresponding column of the underlying response matrix (in this case that in Eq. S1), and applying a classical correction (the bit-flip again) to the result.

The expected probabilities are given by Eq. 6 for this GHZ state and the noiseless probabilities are in the absence of any readout errors. We see that for this GHZ state and readout error profile we only have 8 outcomes in the observed results (as the errors map the two components of the GHZ state to the same set of outcomes). Errors with syndromes in the set $\{0000, 0001, 0110, 0111, 1000, 1001, 1110, 1111\}$ (those observed in calibration) map the outcomes in the observed data for the GHZ state to the same set. This means that we can perform the constrained minimization mitigation while considering only this reduced set of 8 outcomes as opposed to the usual 16.
